# The Association of Resilience with Psychiatric Morbidity, Sleep Quality and Antiretroviral Therapy Adherence among Older Adults Living with HIV in Nigeria

**DOI:** 10.21203/rs.3.rs-11290080/v1

**Published:** 2026-10-08

**Authors:** Olufisayo Oluyinka Elugbadebo, Lisa M. Kuhns, Lauren Brittany Beach, Bibilola D. Oladeji, Chiamaka Egbuna, Olutosin Awolude, Olayinka Omigbodun, Philip B. Gorelick

**Affiliations:** College of Medicine, University of Ibadan, Ibadan, Oyo State, Nigeria; Feinberg school of Medicine & Ann & Robert H. Lurie Children’s Hospital of Chicago, Division of Adolescent and Young Adult Medicine, Northwestern University, Chicago, IL 60611, USA.; Northwestern University Feinberg School of Medicine, Chicago IL, USA; College of Medicine, University of Ibadan, Ibadan, Oyo State, Nigeria; University of Ibadan, Nigeria.; HIV Program, Infectious Disease Institute, University College Hospital, Ibadan, Nigeria.; College of Medicine, University of Ibadan, Ibadan, Oyo State, Nigeria; Northwestern University Feinberg School of Medicine

**Keywords:** Older adults living with HIV, Resilience, Psychiatric morbidity, ART adherence, Depression, Sleep quality

## Abstract

Advances in antiretroviral therapy (ART) have increased survival among people living with HIV, resulting in a growing population of older adults who often face persistent psychological challenges. However, limited evidence exists on how in the face of these challenges, resilience may interact with psychiatric morbidity and HIV treatment adherence in African countries. This study therefore examined resilience among older adults living with HIV in Ibadan, Nigeria and its association with psychiatric morbidity and ART adherence.

In this cross-sectional study, resilience, psychiatric morbidity and HIV treatment adherence were assessed among 400 older adults aged ≥ 50 years living with HIV. Resilience, depression, anxiety and sleep quality were measured using the 25-item Wagnild and Young resilience scale, Patient Health Questionnaire-9 (PHQ-9), Generalized Anxiety Disorder 7-item scale (GAD-7) and the Pittsburgh Sleep Quality Index (PSQI) respectively. Antiretroviral medication adherence was self-reported for the prior 30-days. Associations between resilience levels, psychiatric morbidity, and medication adherence outcomes were investigated using multivariable logistic regression controlling for demographic and clinical factors.

The mean age of participants was 58.2 ± 6.5 years. Low resilience was common, affecting approximately 89.7% of the cohort. The prevalence of psychiatric morbidity was 7.8% for depression, 6.0% for anxiety, and 15.2% for poor sleep quality. Overall, ART adherence was relatively high, with 85.5% of participants reporting adequate adherence. Higher resilience scores were associated with lower odds of depression (adjusted [aOR] = 0.958, 95% CI: 0.939–0.977), anxiety (aOR = 0.971, 95% CI: 0.950–0.993, p = 0.010) and an increased odds of at least 95% antiretroviral therapy adherence (aOR = 1.033, 95% CI: 1.008–1.058, p = 0.009).

In this study of older adults living with HIV in Nigeria, the level of resilience was low but was protective for psychiatric morbidity and medication adherence. Promoting resilience may improve well-being among aging populations living with HIV.

## Introduction

Over the past decades, the scale up of antiretroviral therapy (ART) has significantly reduced HIV-related mortality[1] and transformed HIV from a fatal illness into a chronic manageable condition[2]. As a result, an increasing proportion of people diagnosed earlier in adulthood are now surviving into older age. As survival improves, increasing numbers of people living with HIV are reaching older adulthood. Although major progress has been achieved in viral suppression, immune recovery, and life expectancy,[3] less attention has been paid to the long-term psychological impact of aging and living with HIV[4].

In Nigeria and other parts of sub-Saharan Africa, the population of older adults living with HIV continues to grow. Improved survival in this group reflects the success of ART programmes and expanded access to HIV care[5]. However, older adults living with HIV (OLHIV) often face persistent psychosocial stressors overtime. These include stigma,[6] social isolation,[7] comorbidities,[8] and the burden of life-long medication and treatment[9]. Although many individuals develop coping strategies while managing long-term illness,[10] the psychological demands of living with HIV over decades may gradually affect emotional well-being, even among those who achieve sustained viral suppression[11].

Evidence from African settings suggests that older adults often demonstrate better antiretroviral therapy (ART) adherence than younger populations[12]. This pattern has been attributed to more structured daily routines, greater trust in medical advice, and a heightened perception of ART[13] as essential for survival, especially among individuals who witnessed earlier phases when treatment options were limited;[13] nevertheless, long-term engagement with treatment may also present challenges. Prolonged exposure to ART and the ongoing management of a chronic illness can contribute to treatment fatigue and psychological strain over time[14].

Resilience, the capacity to adapt positively to stress and maintain function, has been identified as an important factor in coping with chronic diseases[15]. Some frameworks focus on resilience as a personal trait, others view it as a dynamic process, while still others emphasize the role of environmental and systemic factors[16]. Resilience has been conceptualized as a personal trait, a dynamic process (or trajectory), or both[17]. As a personal trait, resilience refers to an individual’s relatively stable capacity to adapt, thrive, or recover following adversity or stressful life events[18]. In contrast, resilience as a trajectory is conceptualized as changes in symptoms or functioning over time, reflected by recovery or the maintenance of stable functioning in response to adversity[19–21]. Consequently, resilience measures differ in the aspect they assess. Instruments such as the 25-item resilience scale[22] used in this study measures an individual’s degree of psychological resilience[23]. However, resilience itself is increasingly recognized as a construct that can be strengthened through supportive relationships, adaptive coping strategies, and psychosocial interventions[24].

Low resilience may increase susceptibility to mental illness and inability to cope with daily stressors[25]. Resilience is not a fixed attribute but a dynamic process influenced by personal resources, social support, and life experiences[26, 27]. For individuals aging with HIV, this adaptive process occurs alongside multiple pressures, including multimorbidity, functional decline, persistent stigma, and changes in social roles[28, 29]. When coping resources become strained, vulnerability to mental health conditions such as depression, anxiety, and sleep disturbances may increase. Despite growing recognition of the psychosocial challenges faced by OLHIV, evidence on resilience and its relationship with psychiatric morbidity and treatment adherence remains limited in many low- and middle-income settings. Much of the existing research in the region has focused on virologic outcomes and treatment adherence, with comparatively little attention to the psychological resources that support long-term engagement in care.

The study examined resilience among OLHIV by estimating resilience levels and assessing its association with psychiatric morbidity including depressive symptoms, anxiety symptoms and sleep quality as well as antiretroviral therapy adherence. We therefore hypothesize that higher resilience would be associated with lower psychiatric morbidity and better ART adherence among older adults living with HIV.

## Methods

### Study design and setting

We conducted a cross-sectional study among OLHIV receiving care at a tertiary health facility in Ibadan, Nigeria. The study was conducted at the Infectious Disease Institute (IDI), located in Ibadan, Nigeria. The Institute provides comprehensive HIV services, including diagnosis, initiation and long-term management of antiretroviral therapy (ART), routine clinical monitoring, and management of HIV-related comorbidities. It serves a large and diverse population of people living with HIV, including a growing number of older adults receiving HIV care. The study period was between March and April 2023.

### Study Participants and Eligibility criteria

The study population was comprised of individuals aged ≥ 50 years living with HIV who were identified from the clinic registry and electronic medical records. Eligibility criteria included: (i) participants aged ≥ 50 years; (ii) confirmed HIV infection from clinic records; and (iii) who had at least one clinic visit between January 2019 and December 2023. Individuals with severe cognitive impairment that could limit participation or compromise the validity of clinical data were excluded. Participants were recruited consecutively during routine clinic visits. Eligible patients were approached, the study was explained to them, and those who provided written informed consent were enrolled.

### Sample size

According to the data available at the IDI as of November 2022, the total population of OLHIV who were on ART was 2851. Yamane’s formula for a known population was used to estimate the minimum sample size with CI of 95% and margin error of 0.05 assumed.

The sample size was calculated as follows:

n=N/1+Ne

- *n* =Estimated sample size
- *N* = Number of total population
- *e* = margin of error set at 0.05

$$\frac{2851}{1+2851\;(0.05\;X\;0.05)}=$$

$$\frac{2851}{8.1275}=$$

350.7 ≈ 351

To compensate for non-response rate of 10% (which is ≈ 35),

The estimated sample size is 386, which was approximated to 400

### Variables

The primary outcome of this study was psychiatric morbidity (depression, anxiety, and sleep disturbance). Antiretroviral therapy adherence was examined as a secondary outcome. Resilience was the main independent variable. Sociodemographic and clinical characteristics were included as potential confounders.

### Data Collection Procedure

All study measures were collected as part of a single cross-sectional research survey. Interviews on the participants’ sleep quality, resilience, treatment adherence, depressive and anxiety symptoms were carried out by trained research assistants literate in English and Yoruba and interviewed using a structured questionnaire. This allowed interviews to be conducted in the participant’s language of preference. Interviews were conducted face – to- face with participants in a confidential setting during routine clinic visit using the participant’s preferred language. Each interview lasted approximately 45 minutes on average, and participants were assured that their responses would be kept confidential and used solely for research purposes. Participants were also informed that participation was voluntary and that they could decline to answer any question or withdraw from the study at any time without affecting the care they received at the clinic.

### Data Sources and measurement

#### Psychiatric Morbidity (Primary outcomes)

A composite variable, “any psychiatric morbidity,” was created for analysis. Participants were classified as having any psychiatric morbidity if they screened positive for at least one of the assessed psychiatric conditions (e.g., depression, or anxiety); those who screened negative for all assessed conditions were classified as having no psychiatric morbidity. Psychiatric morbidity was assessed using the Patient Health Questionnaire-9 (PHQ-9) and the Generalized Anxiety Disorder-7 (GAD-7), which measure depressive and anxiety symptoms, respectively:

##### Depression:

Depressive symptoms were measured using the Patient Health Questionnaire-9 (PHQ-9)[30], a 9-item scale assessing symptoms over the past two weeks which includes items such as “Little interest or pleasure in doing things” and “Feeling down, depressed, or hopeless.” Each item is scored from 0 to 3, yielding a total score of 0–27. Scores of 5–9 indicate mild depression, 10–14 moderate depression, 15–19 moderately severe depression, and 20–27 severe depression. A cut-off score of ≥ 10 was used to define clinically significant depressive symptoms.

##### Anxiety:

Anxiety symptoms over the past two weeks were assessed using the Generalized Anxiety Disorder 7-item scale (GAD-7).[31] Items include, “Feeling nervous, anxious, or on edge” and “Not being able to stop or control worrying.” Each item is scored from 0 to 3, with total scores ranging from 0 to 21. Scores of ≤ 5, 6–10, and 11–15 indicate mild, moderate, and severe anxiety, respectively. A cut-off score of ≥ 10 was used to define clinically significant anxiety.

##### Sleep Quality:

Sleep quality was assessed using the Pittsburgh Sleep Quality Index (PSQI)[32], a 19-item instrument that evaluates sleep quality over the previous month. It asks questions such as “During the past month, when have you usually gone to bed at night?” and “How would you rate your overall sleep quality?” The global PSQI score ranges from 0 to 21, with higher scores indicating poorer sleep quality. A global score ≥ 5 was used to define poor sleep quality.

#### Resilience (Independent variable)

Resilience was assessed using the 25-item Wagnild and Young Resilience Scale (RS-25)[33], which measures five core characteristics of resilience which were divided into two domains: personal competence, reflecting self-efficacy, determination, self-reliance, and ability to manage competing demands (e.g., “When I make plans, I follow through with them” and “I usually manage one way or another”); and acceptance of self and life, reflecting adaptability, self-acceptance, optimism, and the ability to maintain a balanced perspective and cope with adversity (e.g., “I usually take things in stride” and “I can be on my own if I have to”). Each item is rated on a 7-point Likert scale (1 = strongly disagree to 7 = strongly agree), with total scores ranging from 25 to 175. Higher scores indicate greater resilience. For analytical purposes, all items were analyzed together as a single composite score and resilience levels were categorized into low resilience (25–130) and ≥ 131 as high resilience representing at least moderate resilience according to Wagnild’s interpretive guidelines[34].

##### Treatment Adherence

Adherence to ART was assessed using self-reported medication use over the preceding 30 days. It included questions on the frequency of missed doses and adherence to prescribed medication regimens. Adherence was dichotomized as optimal or suboptimal, with suboptimal adherence defined as taking less than 95% of prescribed doses[35].

#### Covariates and Potential Confounders

Sociodemographic variables (age, sex at birth, highest level of education, and marital status) were self-reported while the clinical characteristics (duration of HIV infection, duration on ART, comorbid conditions - hypertension, renal impairment, dermatitis, and arthritis) were extracted from the clinical records. These variables were considered potential confounders in the analysis. These variables were selected based on prior literature [36] and their known associations with both resilience and mental health outcomes[37].

##### Bias

To minimize selection bias, consecutive recruitment of eligible participants was employed. Information bias was reduced using standardized and validated instruments administered by trained research personnel. Confounding was addressed using multivariable regression analyses.

### Statistical Analysis

All data were entered into and analyzed using SPSS Statistics Version 27[38]. Descriptive statistics were carried out to summarize participants’ demographic and clinical characteristics. Continuous variables were summarized using means and standard deviations, while categorical variables were presented as frequencies and percentages. Age was categorized as 50–59, 60–69, and ≥ 70 years; sex at birth as male or female; marital status as married or unmarried (including single, divorced, and widowed); religion as Christian or Islam; educational status as no formal education, primary, secondary, or tertiary education; and living condition as living alone or living with others. Clinical characteristics, including age at diagnosis was categorized as 27–37, 38–48, 49–59, and 60–70 years, while year of diagnosis was categorized as 1997–2006, 2007–2016, and 2017–2021. Duration of HIV infection and duration on ART were treated as continuous variables. ART adherence was categorized as ≥ 95% (Yes) vs < 95% (No). A composite comorbidity variable was created to indicate the presence of at least one documented comorbid condition (hypertension, renal impairment, dermatitis, or arthritis). Comorbidity was categorized as Yes or No based on the presence of any recorded comorbidity.

Multivariable logistic regression models were used to estimate odds ratios (ORs) and 95% confidence intervals (CIs) for the association between resilience and depression, sleep quality, and suboptimal adherence. Models were adjusted for age, sex at birth, education status, duration of HIV infection, duration on ART, and comorbid conditions. The primary exposure was resilience, and participants were stratified into low- and high-resilience groups. Outcomes included Psychiatry morbidity (depression, anxiety) sleep quality, and ART adherence depression and anxiety were categorized as Yes or No, sleep quality as poor or good, and ART adherence as optimal (≥ 95%) or suboptimal (< 95%). Participants were classified as having any psychiatric morbidity if they screened positive for either depression (PHQ-9 ≥ 10), anxiety (GAD-7 ≥ 10), or both. This composite binary outcome (yes/no) was used in the analyses of psychiatric morbidity.

Statistical significance was set at p < 0.05. Data completeness was assessed prior to analysis, and no missing data were identified for the variables analyzed. This study is reported in accordance with the STROBE (Strengthening the Reporting of Observational Studies in Epidemiology) guidelines.

## Results

### Participant Characteristics

A total of 400 older adults living with HIV were assessed. The mean age of the participants was 58.2 years (± 6.5). Women represented a greater proportion of the sample (n = 254; 63.5%) by birth sex. Regarding living conditions, 81.5% of the respondents resided with others, while the remaining 18.5% lived alone. A majority reported having at least one comorbid condition and 63.2% were diagnosed with HIV between 2007–2016. (Table 1)

### Clinical Characteristics

The prevalence of low and high resilience was 89.7% and 10.3%, respectively (see mean item scores, Supplementary Table 1). Suboptimal ART adherence (< 95% of prescribed doses) was reported by 14.5% of the participants. Participants had a mean duration of 12.3 years (SD = 4.4) on ART and a mean duration of 13.1 years (SD = 4.5) since HIV diagnosis. Psychiatric morbidity (depression and/or anxiety) was found in 13.8% of the participants with 84.8% of the participants having good sleep quality and 43% having comorbidity (Table 2)

### Logistic regression

In multivariable logistic regression, adjusting for sociodemographic and clinical covariates, resilience was a significant predictor of any psychiatry morbidity (aOR = 0.966, 95% CI: 0.937–0.996, p = 0.028). More specifically, resilience was associated with lower odds of depression (adjusted [aOR] = 0.962, 95% CI: 0.932–0.993, p = 0.016) and lower odds of anxiety (aOR = 0.971, 95% CI: 0.950–0.993, p = 0.010), but not poor sleep quality (aOR = 0.997, 95% CI: 0.957–1.039, p = 0.896). Furthermore, resilience was significantly associated with increased odds of optimal ART adherence (aOR = 1.033, 95% CI: 1.008–1.058, p = 0.009). Demographic and clinical factors were not significantly associated with either primary or secondary outcomes, except that participants with formal education had lower odds of poor sleep quality compared to those with no formal education (aOR = 0.230, 95% CI: 0.067–0.789, p = 0.019).

## Discussion

This study examined resilience and its association with psychiatric morbidity, sleep quality, and ART adherence among OLHIV at a clinic in Ibadan, Nigeria. Low resilience was common in this population. Resilience was associated with lower odds of depression and anxiety and better ART adherence. These findings suggest that resilience is an important factor related to both psychological well-being and ongoing engagement in care among OLHIV.

The low level of resilience observed in this cohort highlights potential vulnerability among older adults living with HIV in this setting. This may have implications for mental health outcomes, as lower resilience has been linked with poorer psychological well-being[39]. Although ART has improved survival and HIV disease control, many individuals living with HIV continue to live with persistent psychosocial and clinical burdens. OLHIV frequently experience stigma, social isolation, and multiple chronic health conditions,, all of which may influence emotional well-being over time[40, 41]. Previous work on HIV and ageing has similarly highlighted the layered challenges faced by this population, including multimorbidity, psychosocial challenges and long-term treatment burden[42, 43]. These challenges often accumulate across the life course and may gradually reduce coping capacity, increasing vulnerability to mental health conditions. Consistent with this, previous studies have reported higher rates of depression and anxiety among people living with HIV compared with the general population [44, 45].

The association between resilience and psychiatric morbidity in this study aligns with existing literature. Resilience has been conceptualised as a trait, a dynamic process, or both [17]. Although closely related, resilience is distinct from mental health, coping, and psychiatric morbidity. While mental health reflects overall psychological well-being and psychiatric morbidity refers to clinically significant mental health symptoms or disorders, coping describes the strategies used to manage stress. Rather than representing the absence of mental illness, resilience reflects the capacity to adapt positively to adversity through personal and social resources that may reduce vulnerability to psychiatric disorders and extends beyond coping by encompassing the broader personal and social resources that enable successful adaptation despite adversity.

Resilience has been described as the capacity to adapt positively to change and adversity and maintain psychological functioning in the face of stress [46, 47]. Individuals with higher resilience tend to demonstrate more effective coping strategies and better emotional regulation when faced with chronic illness [48]. Among people living with HIV, resilience has been linked to lower levels of depressive symptoms and improved psychological well-being [49, 50]. The present findings reinforce this relationship in an older cohort, suggesting that resilience remains relevant even after many years of living with HIV. Resilience was not significantly associated with sleep disturbance, thus, this study did not provide evidence to support that hypothesis.

Furthermore, resilience was associated with better ART adherence. Adherence in older adults is influenced not only by access to care but also by the ability to sustain routines, manage competing demands, and respond to stress[51]. Resilience may support these processes by reinforcing persistence and maintaining treatment behaviours, despite ongoing challenges. This is consistent with prior evidence showing that resilience is associated with improved adherence and viral suppression [52, 53].

At the same time, psychiatric morbidity remains an important barrier to optimal treatment outcomes. Drawing on the stress and coping theory,[54] depression and anxiety may weaken coping resources and reduce motivation, concentration, and day-to-day self-management. Social cognitive theory[55] also helps explain this relationship, as adherence depends partly on self-efficacy and self-regulation. Evidence from a meta-analysis shows that depression reduce the odds of high ART adherence by over 40%[56]. In this context, resilience may act as a buffer, reducing the impact of psychological distress on treatment behaviour[57]. The relationship between resilience, mental health, and adherence is therefore likely to be interconnected rather than independent[58, 59].

These findings have implications for HIV care in ageing populations, particularly in low-resource settings. Much of the focus of HIV programs has been on virologic outcomes and treatment delivery, with less attention to the psychological factors that support long-term engagement in care[60, 61]. Although resilience is measured as a relatively stable resource in this study, resilience theory suggests that it may be strengthened through interventions that enhance individual, social and environmental resources. Findings from our study suggest that mental health and resilience outcomes should be considered as important, alongside virologic indicators. Routine screening for depression and anxiety, together with psychosocial interventions that support adaptive coping, social support and resilience may help to improve both psychological well-being and treatment outcomes in older adults living with HIV. Although, evidence specifically among older adults remain limited, systematic reviews[62] suggests that psychosocial interventions including cognitive-behavioural therapy, peer-support programs and multi-component psychosocial interventions can enhance resilience and psychological well-being in people living with HIV and older adults, supporting resilience as a potential target for future intervention research[62, 63].

Several limitations should be considered when interpreting these findings. First, resilience, psychiatric morbidity, ART adherence, were assessed at a single time point. Consequently, the temporal direction of the observed associations cannot be determined. Second, HIV treatment adherence was assessed using self-reported measures, which may be subject to recall bias. Third, the study was conducted in a single clinical setting, which may limit generalizability. In addition, the study measures were based on different assessment periods. While resilience was assessed as participants’ perceived resilience at the time of survey administration using the study tool, ART adherence reflected the previous 30 days, and other outcomes had their respective assessment periods. These different time frames may have influenced the observed associations. In addition, although resilience is now widely viewed as a dynamic and modifiable process, the 25-item RS scale primarily captures resilience as a relatively stable characteristic at a single point in time.

In conclusion, low resilience was common among older adults living with HIV and was associated with depression, anxiety, and ART adherence. These findings underscore the need to address psychological as well as clinical dimensions of HIV care as this population continues to age.

## Supplementary Material

Supplementary Files

This is a list of supplementary files associated with this preprint. Click to download.

- SupplementaryTablesTheAssociationofResilience.docx

## Figures and Tables

**Table 1:** Socio-demographic characteristics of participants

Variables	N	Percentage (%)
**Age**		
50–59	257	64.3
60–69	115	28.7
70 and above	28	7.0
Mean (± S.D)	58.2	6.5
**Age at diagnosis**		
27–37	57	14.2
38–48	233	58.3
49–59	90	22.5
60–70	20	5.0
**Sex at birth**		
Male	146	36.5
Female	254	63.5
**Marital Status**		
Unmarried (e.g., currently single, divorced, widowed)	180	45.0
Married	220	55.0
**Religion**		
Christian	277	69.2
Islam	123	30.8
**Educational status**		
No formal education	24	6.0
Primary education	95	23.8
Secondary education	135	33.8
Tertiary education	146	36.4
**Present Living Condition**		
Living alone	74	18.5
Living with others	326	81.5
**Year of Diagnosis**				
1997–2006	104	26.0
2007–2016	253	63.2
2017–2021	43	10.8

**Table 2:** Clinical characteristics of the participants

Characteristics	Value
Duration on ART (mean, SD)	12.3yrs (4.4)
**ART Adherence (≥ 95%)**
Yes, *N (%)*	342 (85.5)
No, *N (%)*	58 (14.5)
Duration of HIV infection (mean, SD)	13.1yrs (4.5)
**Resilience (mean, SD)**	80.4 ±11.2
Low Resilience	359 (89.7)
High Resilience	41 (10.3)
**Depression**
Yes, *N (%)*	31 (7.8)
No, *N (%)*	369 (92.3)
**Anxiety**
Yes, *N (%)*	24 (6.0)
No, *N (%)*	376 (94.0)
**Sleep Quality**
Poor sleep quality *N (%)*	61 (15.2)
Good sleep quality, *N (%)*	339 (84.8)
**Comorbidity**
Yes, *N (%)*	172 (43.0)
No, *N (%)*	228 (57.0)

**Table 3:** Associations between resilience and psychiatric morbidity among people living with HIV

Resilience	Psychiatric morbidity Odds Ratio (95% CI), p-value	Sleep quality Odds Ratio (95% CI), p-value	Depression Odds Ratio (95% CI), p-value	Anxiety Odds Ratio (95% CI), p-value	ART Adherence Odds Ratio (95% CI), p-value
	**0.966 (0.937–0.996), 0.028** *	**0.997 (0.957–1.039), 0.896**	**0.962 (0.932–0.993), 0.016** *	**0.971 (0.950**–0.993), 0.010*	**1.033 (1.008–1.058), 0.009** *
**Age**	0.968 (0.916–1.024), 0.254	1.058(0.944–1.126), 0.076	0.987 (0.930–1.047), 0.658	0.937 (0.865–1.015), 0.112	0.968 (0.927–1.010), 0.133
**Sex at birth**	1.254 (0.590–2.665), 0.556	1.871 (0.672–5.207), 0.230	1.753 (0.764–4.023), 0.185	0.804 (0.316–2.044), 0.646	10.903 (0.486–1.679), 0.748
**Formal education (primary or higher)**	0.755 (0.230–2.476), 0.642	0.230 (0.067–0.789), 0.019*	0.868 (0.244–3.093), 0.827	2.389 (0.289–19711), 0.419	0.000
**Duration of HIV infection**	0.810 (0.601–1.092), 0166	0.857 (0.569–1.289), 0.458	0.851 (0.617–1.175), 0.327	0.629 (0.384–1.029), 0.065	0.857 (0.569–1.289), 0.458 0.942 (0.801–1.108), 0.471
**Duration on ART**	1.145 (0.905–1.450), 0.260	1.133 (0.802–1.599), 0.480	1.150 (0.889–1.488), 0.286	1.395 (0.878–2.217), 0.159	1.052, (0.897–1.234), 0.534
**Comorbid conditions**	0.812 (0.397–1.661), 0.568	0.742 (0.293–1.883), 0.530	0.735 (0.341–1.586), 0.432	1.069 (0.439–2.604), 0.883	1.109 (0.621–1.981), 0.727

* Psychiatry morbidity- risk of either depression, anxiety or both; *Comorbid conditions- includes physical/medical condition such as hypertension, renal impairment, dermatitis, or arthritis

* Variable significant at < .05

## Data Availability

The data used in this study is not publicly available but may be made available to qualified researchers on reasonable request *from the corresponding author*.

## Code Availability

The underlying code for this study is not publicly available but may be made available to qualified researchers on reasonable request from the corresponding author.
